# Bi^5^: An Autoethnographic Analysis of a Lived Experience Suicide Attempt Survivor Through Grief Concepts and ‘Participant’ Positionality in Community Research

**DOI:** 10.3390/socsci14070405

**Published:** 2025-06-26

**Authors:** amelia elias noor

**Affiliations:** Bloomberg School of Public Health, Johns Hopkins University, Baltimore, MD 21205, USA

**Keywords:** lived experience, participatory research, trauma-informed, gender-sensitive research

## Abstract

This paper explores suicidality and suicide research from an autoethnographic analysis framed through grief concepts. Self-identifying as a Muslim in the United States, the author explains how lived experiences being racialized through Islamophobia, identifying as a genderfluid non-binary woman, being socially biracial, holding a postpartum bipolar diagnosis, and being connected to a diaspora, are critical elements to develop a deeper sociocultural understanding of suicide. Grief concepts that are used to analyze these themes include disenfranchised grief, ambiguous loss, anticipatory grief, and secondary loss. While these grief concepts are understood as part of the author’s embodied lived experience as an individual, there is also a collective grief that is explored through the author’s bilingual experience with Arabic as it relates to the topics of suicide and genocide occurring in the Arabic-speaking diaspora located in Gaza, Palestine. A conceptual framework is offered to make sense of the author’s lived experience by both incorporating and challenging existing academic perspectives on suicide and research. The *emic*, or insider, perspective is contextualized such that it may hold implications beyond the individual author, such as for U.S. Muslims and other hard-to-reach populations. A positionality statement demonstrates the author’s reflexivity of being an insider ‘participant’–researcher in conducting transformative research approaches with the U.S. Muslim community. Further directions are shared for scholars with lived experience who may seek to utilize comparable individual or collaborative autoethnographic approaches with such majority-world communities.

The Prophet Muhammad (ﷺ --peace and blessings upon him) said:

“Let none of you wish for death because of a calamity that has befallen him. But if he must wish for something, let him say: ‘O Allah, keep me alive as long as life is better for me, and cause me to die when death is better for me.’”

—[Sahih al-Bukhari 5671, Sahih Muslim 2680]

## 1. Introduction

Within the field of social and behavioral sciences, human subjects research may draw from anthropological, cultural, and socioecological foundations, such as through the use of ethnographic approaches. For example, topics like social norms and their effects on individual and group behavior exist in the research literature on public health equity (Horrill et al. 2024), including suicide which is defined as intentional self-targeted violence resulting in an injury causing premature death (Staples 2015; Cetin 2016; Staples and Widger 2012). Ethnography is a qualitative research method that involves immersive observation and sustained engagement with a group or community over time, typically with the aim of understanding the community’s daily practices, cultural values, and shared worldviews from the perspectives of its members. This approach frequently includes both direct participation in and observation of communal life, generating what Geertz (1973) termed “thick description”—rich, contextualized accounts of behavior and meaning-making processes.

A central consideration in ethnography is the researcher’s positionality in relation to the community under study, often framed in terms of emic and etic perspectives (Markee 2012). The *emic* perspective refers to an insider’s view—how members of a group understand and interpret their own beliefs, values, and behaviors from within their cultural framework. In contrast, the *etic* perspective involves an outsider’s analytical lens, using concepts that may not originate from within the group but allow for a comparison or interpretation across other contexts. Both perspectives can be complementary; the emic offers depth and authenticity, while the etic provides an ecological validity, or generalizability, to other social groups (Morris et al. 1999). Understanding the differences in emic and etic approaches is especially important for scholars conducting qualitative research with decolonial practices that are meant to be transformative or liberatory for the groups being studied (Thambinathan and Kinsella 2021).

Autoethnography is an ethnographic method that conveys a qualitative account of the researcher as participant, using embodied inquiry, or lived experience with one’s body and mind (Scott-Pollock et al. 2022). The method of autoethnography combines elements of autobiography and ethnography and reflects both a process and product of the writer making meaning of their lived experiences (Ellis et al. 2011). It involves the author as simultaneously a participant and researcher, detailing personal narratives that are situated within the broader cultural experiences of the group or community being studied (Ellis et al. 2011). An autoethnography is, according to Ellis et al. (2011), a research and writing approach that serves as a form of reflexive ethnography, in which the boundaries between the roles of ‘researcher’ and ‘participant’ are fluid, and reflect deep self-examination that illuminates shared experiences (Ellis et al. 2011).

Historically, feminist and Black scholars such as bell hooks characterized the personal as the political and emphasized the importance of narratives centered in intersectional lived experiences to disrupt the dominant and often negative portrayals of oppressed populations. Therefore, the purpose of autoethnography is to describe and analyze lived experience such that its meaning is interpreted in the context of bringing a greater understanding of the “connectivity between self and others within the same context” (Ngunjiri et al. 2010, p. 1). The method of autoethnography was popularized more recently by Carolyn Ellis as “a reflexive approach to research, writing, and storytelling that connects the autobiographical and personal to the cultural, social and political” (Carolyn Ellis | Communication Department | USF n.d.) “Ethnographic analysis is not exclusively emic. Rather, a carefully done emic analysis precedes and forms the basis for etic extensions that allow for cross-cultural or cross-setting comparisons” (Watson-Gegeo 1988, pp. 580–81). The objective of this autoethnography is to relay certain lived experiences from an insider, or emic, perspective. In this research, my role as a self-identifying Muslim studying U.S. Muslim communities reflects an emic positionality, granting me cultural familiarity, linguistic access, and experiential insight that shapes both data collection and interpretation. At the same time, I remain committed to reflexively engaging with etic frameworks from social science to analyze broader patterns and situate my personal narratives within the wider scholarly discourse regarding community and population health. In doing so, one of my aims is to promote relatability to the reader through anecdotes that make up parts of my life story centered around narratives of grief involving themes of xenophobia, gender, racialization, healthcare, language, and faith.

Note that grieving is broadly acknowledged as an evolving process in which meaning can be reconstructed over time, and usually involves the death of something or someone that may be tangible or intangible (Barney and Yoshimura 2020). In this autoethnography, the context of grief is meant to connect my personal life stories with others’ who identify as Muslim, and even those who may not. Additionally, the reader may or may not hold a background in experiencing suicidal thoughts or behaviors (STB) yet may hold shared lived experiences with certain spiritual or emotional aspects of meaning-making in the grieving process. The relatability of this meaning-making process between me and other members of the U.S. Muslim community is explored further in the discussion on the topic of collaborative ethnographies for future research directions.

### 1.1. Significance: Theory, Methodology, and Demography

A key significance behind this autoethnography of a self-identifying Muslim suicide attempt survivor is to disrupt dominant narratives of Muslims situated in the Global North (Fanon 1961; Shahwar 2014; Samari 2016; Beydoun 2017), and also to help inform future policy promoting mental health equity. In the United States, for example, there is a long sociopolitical history targeting Muslims through institutionalized Islamophobia (Levin and Idler 2018). This anti-Muslim ideology has fueled stereotypic tropes of Muslims as a violent and homogenous group, effectively racializing the adherents of the Islamic faith into a socially stigmatized “other” (Garner and Selod 2015). The otherization of Muslims in the Global North has led to increased reports of discrimination and intrapersonal violence, i.e., suicide, such as in the United States where a study revealed double the rate of a lifetime history of suicidal thoughts or behaviors in Muslims compared to other faith groups (Awaad et al. 2021). Research on the health effects of Islamophobia continue to indicate trends of poorer mental health in U.S. Muslims, suggesting the how anti-Muslim social stigma as a normative ideology becomes internalized within the individual. My individual account offers a unique contribution to the literature on suicide research broadly, and to Muslim mental health, specifically.

From a theoretical perspective, this autoethnography was heavily inspired from concepts established by previous scholars such as Chandra Ford, Ilan Meyer, and Mark Hatzenbuehler. Ford’s Public Health Critical Race Praxis offers a framework for understanding how racism—and by extension, Islamophobia—can be structurally embedded in health research, policy, and practice (Ford and Airhihenbuwa 2010). It emphasizes the importance of centering marginalized voices and lived experiences in public health inquiry, which aligns with this study’s methodological emphasis on autoethnography as both personal and political. Similarly, Meyer (1995)’s Minority Stress Theory, and Hatzenbuehler (2016)’s work on stigma mechanisms, provide insight into how chronic exposure to discrimination, social rejection, and identity-based stigma contribute to mental health disparities among minoritized populations (Meyer 1995; Hatzenbuehler 2016; Noor-Oshiro 2021). Applying these frameworks to Muslim communities allows for a deeper understanding of how macro-level ideologies like institutionalized Islamophobia can become internalized and manifest as psychological distress, including suicidal ideation. My narrative, then, is not merely anecdotal—it is situated within a broader socioecological context, where the intersections of religion, race, and stigma shape life and death outcomes. This account builds on and contributes to existing theories by offering an emic and embodied attestation of what it means to survive within systems that mark Muslim lives as suspect, disposable, or invisible.

Said’s (1978) foundational work *Orientalism* is directly relevant to my autoethnography, as it exposes how Western knowledge production has historically constructed the “Orient” (including Islam and Muslims) as backward, irrational, exotic, and dangerous in contrast to the Western notions of modernity and superiority (Said 2016). Instead, my narrative directly challenges these dominant tropes by reclaiming my voice and lived experiences of suicidal thoughts and behavior as a direct result of Western imperialism. Said’s work inspires my understanding of how self-targeted violence is linked to systems of historical colonial violence, imperialism, and surveillance. By situating my personal experiences of suicide survival, mental health stigma, and Islamophobia within broader historical patterns of misrepresentation and marginalization, I resist being positioned as an object of study and instead assert myself as a producer of knowledge. Hence, Said’s critical stances not only inform the theoretical grounding of my autoethnography but also legitimize it as a form of political resistance.

Methodologically, my use of autoethnography is deeply informed by the principles of decolonial and transformative qualitative research established by previous scholars. There is an emphasis on recognizing that traditional Western research paradigms often reproduce colonial hierarchies of knowledge by privileging Eurocentric epistemologies while marginalizing Indigenous and non-Western ways of knowing. In response, my narrative—centered on the lived experience of being a Muslim suicide attempt survivor in the U.S.—represents an intentional act of epistemic resistance. As Thambinathan and Kinsella (2021) argue, decolonizing qualitative research requires creating space for critical reflexivity, relational ethics, and the validation of “other(ed)” ways of knowing (Thambinathan and Kinsella 2021). My autoethnographic approach takes up this challenge by positioning my body and story as a legitimate source of knowledge and critique, one that is situated within broader structures of Islamophobia, racialization, and postcolonial trauma. In line with Browne et al.’s (2005) call to challenge health research practices that erase or pathologize marginalized communities, my method shows a sense of self-determination, while contextualizing the structural and sociopolitical contexts that shape my psychological distress. In this way, autoethnography becomes a combined method and praxis in that I seek to not only produce knowledge but also to contribute to healing, visibility, and collective transformation for the U.S. Muslim community.

Furthermore, as a self-identifying Muslim raised and residing in the Global North, my autoethnographic reflection is shaped by a consciousness of how colonial legacies continue to define whose life matters. Postcolonial theory challenges dominant Western frameworks of knowledge and instead calls attention to the imperial power dynamics embedded in knowledge production and representation. Scholars Browne et al. (2005) emphasize that postcolonial perspectives are especially critical in health research with historically oppressed communities, like indigenous and aboriginal, as they expose how racialized and colonized bodies have been pathologized, misrepresented, or erased by biomedical paradigms that ignore social and structural dimensions of health (Browne et al. 2005). In my case, the postcolonial lens allows for an interrogation of how the Muslim body is both surveilled and silenced within U.S. sociopolitical discourse—rendered simultaneously hyper-visible through public scrutiny yet invisible through public health neglect. This theoretical grounding makes space for a narrative that resists dehumanizing tropes of Muslims in the Western gaze and reclaims storytelling as a form of epistemic agency and survival. Thus, my lived experience may be explained through a synergistic mix of scientific theories that span critical race praxis (Ford and Airhihenbuwa 2010), structural stigma as a fundamental cause of health inequities (Hatzenbuehler 2011; Duncan and Hatzenbuehler 2014; Hatzenbuehler et al. 2009); interpersonal theory of suicide, and particularly, issues with belongingness (Van Orden et al. 2010); minority stress theory (Meyer 1995); intersectionality theory (Bowleg 2012); historical trauma (Sotero 2006); the socioecological model (Standley 2022); life course theory (Elder 1998); and postcolonial theory, given that most countries, Muslim or otherwise, are historically and some presently colonized (Browne et al. 2005). Relatedly, regarding the demographic significance, the vast majority of U.S. Muslims are a minority racial or ethnic background, and over 70% are first- or second-generation immigrants (Demographic Portrait of Muslim Americans 2017) who are often multilingual. Second-generation immigrants of various racial/ethnic and religious backgrounds have been found to be at elevated risk of suicide in certain parts of the Global North (Forte et al. 2018), demonstrating urgency in developing suicide prevention strategies in both the domestic and global Muslim diaspora. Muslims comprise 1% of the total U.S. populace and are a hard-to-reach population in which community norms influence help-seeking behaviors and underreporting suicide (Amri and Bemak 2013). People of migrant origin will account for 88% of U.S. population growth through 2065, and by 2050, 93% of the growth of the U.S. working-age population will be accounted for by immigrants and their children (Radford n.d.; Pew Research 2013). Globally, by 2030, Muslims will account for roughly three in ten people (29.1%) aged 15–29 in the world—the same age group in which suicide is a top four leading cause of death worldwide (Lugo and Cooperman 2011). Hence, Muslims domestically and globally are a critical group in which to conduct suicide prevention research. Many migrants—Muslim or not—acculturate to dominant groups that do not look like them nor speak their native language. Hence, I thought that if there was a way to connect with such diverse groups of people, perhaps it would be through shared lived experiences of grief—especially as previous critical suicide scholars have done so in the past (Cesar Riani Costa and White 2024; Sjoblom et al. 2022; Chandler 2019).

### 1.2. Background and Context

Research centering lived experience in investigators who conduct human subjects research is uncommon, unconventional, and sometimes perceived as ‘unscientific’ by positivists. I believe lived experience from an insider emic perspective offers a shift in paradigm to advance the science of suicide prevention through interdisciplinary community-based participatory research approaches. In this section, I aim to provide a more granular insight into what led me to complete an autoethnography through a brief explication connecting my personal timeline and journey to anti-violence research.

In 2015, when I first began graduate studies in public health, my intention was to serve the global Muslim community somehow. At that time, I did not make peace with my initial experiences of suicidality. Thoughts of suicide first occurred to me the day after my marriage in 2014, but they did not feel familiar, so I did not recognize them as suicidal. In hindsight, I would describe that suicidality as a sense of hopelessness or a feeling in my gut that I did not want to live life a certain way. Over the course of the past ten years, and through the course of my marriage, I focused on developing an academic understanding of violence to help me make sense of my lived experiences. I made the *niyyah*, or intention, to commit myself to suicide prevention as a form of worship—we are taught as Muslims that any act can be considered worship when the intention is set first.

It was in 2020 when I decided to formally submit a grant to the National Institutes of Health (NIH) on the topic of preventing suicide in minority communities. What I found most challenging was to try and craft an argument for preventing suicide in U.S. Muslims that would satisfy certain biomedical criteria, namely empirical data on inequities of life expectancy among the U.S. Muslim community. At that point, I submitted a grant framing the intersectionality of racism, nativism, and Islamophobia to establish a theoretical argument on suicide in Asians, adding some anecdotal evidence, and statistical findings on Muslim suicides as an alarming subgroup. One of the chief concerns I had was how to write and pitch to the NIH avoiding a deficit-based orientation.

Over the next year, a study led by Dr. Rania Awaad and Dr. Hamada AlTalib offered initial data on suicide in U.S. Muslims. The study revealed that Muslims in the United States report twice the odds of a lifetime suicide attempt compared to other faith groups (Awaad et al. 2021). Several studies corroborate inequities in mental health for U.S. Muslims, particularly in early adulthood (Abelson et al. 2020). As a Muslim myself, and as a researcher, with lived experience, I finally felt seen. I felt validated. And I felt compelled to carry out research on preventing violence against Muslims, including violence that we target toward ourselves. However, I developed the sense that biomedical research agencies only cared about positivist approaches, methodologies, and epistemologies.

Since Muslim identity is not collected in many prominent national health databases, it became even more evident to me that I would encounter many barriers in conducting suicide prevention research for my community. Beyond this systemic challenge to data collection, I also observed interpersonal challenges in discussing the taboo topic of suicide among my peers in the Muslim community. Right before the pandemic, I decided to boldly “come out” as a lived experience survivor on social media with the goal of incrementally increasing exposure to this topic among my social networks. My main objective was to provide psychoeducation on the topic of suicide. I also shared about my postpartum diagnosis of bipolar.

On social media, in November 2019, I began the channel “@muslim.suicide.researcher” so that I could disseminate what I learned in my graduate program and my experiences in the healthcare system to the public. There were many questions I grappled with—would this interfere with conducting my research in an ‘objective’ manner? Would this backfire in any way, with unintended consequences such as iatrogenic risk? Would I be broaching a topic that was often treated with traditional spiritual interventions with my “scientific” views? Ultimately, I felt that there would be more benefits than risks to sharing knowledge on suicide research online, especially if it meant equipping people with the vocabulary to make meaning or sense of their own lived experiences.

With that in mind, I continued to develop as a research scientist, but in a very different way than I expected. Instead of joining suicide research projects that would provide me with training in biostatistical analysis or a background in epidemiology, I decided to create a path that I felt addressed a pressing gap—a path that I had wished to see in academic research. After realizing that the demographic profile of U.S. Muslims is a microcosm of the near-future American landscape, I figured the most impactful project I could produce to contribute to public health would be to focus on original research with the U.S. Muslim community.

While my grant proposal drew on a participatory concept-mapping method and individual interviews, the pandemic forced me to pivot and replace the original design. For some time, I found it difficult to figure out how to collect data on suicide in U.S. Muslims within the constraints of being virtual, though this turned out to be a big blessing in disguise. When I reached out to my three partnering community-based organizations, we held discussions on what questions were important to ask, and how we would ask these through anonymous survey responses. This resulted in an original ethics-board-approved research project, “U.S. Muslim Community Study of Mental Health,” in which 111 participants were recruited by partnering nationwide with MSAW, FYI, and QC (acronyms for anonymity purposes).

Now, in 2025, after thirteen years’ worth of developing trust across three different Muslim-serving community-based organizations, and by utilizing social media strategies for education and recruitment, I realized how much my positionality played a role. Once it became known that I was an attempt survivor, I would find myself finding deeper meaning in going to ordinary events such as my weekly Saturday Quran class at the local mosque, which I attended regularly from 2021–2023, and periodically thereafter until 2025, resulting in my Arabic memorization of a few chapters of the Quran. Slowly, it became clear that my embodied lived experience as a survivor no longer went unnoticed by Muslim community leaders, congregants, and peers.

After attending many congregational services, I began to strategize how to best make use of my embodied lived experience for the purpose of earning the trust of the community to conduct suicide prevention research. A boundary that became important for me, then, was to travel away from my home community in central Maryland to one that was in Washington, DC, about 30 miles away. Personal boundaries allowed me to be more purposeful and intentional with the professional goals I had in mind, but—in reality—I admittedly always wore both hats. This caused me a great deal of distress as I wondered how to define an ethical distance between me and community members. Even as I endured post-separation abuse, I could not reach out to my sisters for help emotionally for a while, as I felt that keeping my distance as a professional was critical.

Eventually, I began participating in community events and programming that had nothing to do with suicidality or mental health, while also attending some that were directly specific to death. For example, during 2023–2024 I attended multiple sessions of a year-long Sisters’ Circle spiritual meetup on the theme of death, engaging in small group discussions with Muslim women, allowing me to better understand spiritual and social views toward death—not to mention that it was led by Dr. ZS of the Institute for Islamic, Christian, and Jewish Studies (ICJS), who held lived experience as a recent loss survivor. A few years later, I underwent training to learn a traditional protocol for cleaning the bodies of deceased Muslims to prepare for religious Islamic burial from an organization called ‘Bury Me Muslim.’ Additionally, I participated in a 12-month spiritual abuse support group lead by a Black Muslim psychologist and South Asian therapist through a community organization serving adult U.S. Muslims. From 2023–2024, attending these types of programs as a *participant* offered me a deeper insight into both my community and my identity.

Being a participant and researcher—simultaneously—was a positionality I could not find in conventional research approaches. Based on what I found, there were emerging trends in suicide research that centered lived experience, but there was no framework for providing guidance on ethical issues of participating in human subjects’ research as a researcher from a ‘participant’ positionality. Many of the events I attended felt engaging, yet I struggled with how to best convey the importance of suicide research for the Muslim community, both locally and beyond. However, I now view suicide research as not just my work, but the collective work of society, as a communal responsibility to disrupt systems of violence.

Thus, a fundamental reason that I write this autoethnography is to document my lived experience in such a way that I can now convey what I could not convey before, making it accessible and perhaps relatable and eventually actionable, *Inshaa Allah* (God willing). In lay audiences, understanding suicide from a lived experience perspective may be more effective than delivering research presentations on it. There are some aspects of lived experiences that are universal in nature, and it is the aim of this article to demonstrate this dynamic from a perspective that may paradoxically seem niche. I should advise, to any *insider* reader, that it is my intention to defer to the indigenous Islamic ways of understanding suicide from a spiritual perspective, yet I am not qualified to offer this yet. Hence, my academic training in social and behavioral sciences and my research background in social dimensions of health better frames this autoethnography.

### 1.3. On Grief

Grieving is often overlooked in therapeutic, research, and clinical encounters, based on my observations. Often, in my experiences with the Muslim community and outside of it, I found that people did not have the terminology to make sense of their lived experiences, including myself. When I attended the Muslim Mental Health conference in 2022, a couple of Muslim therapists led a workshop on four concepts of grief, which impacted me in a profound way. A year later, I began to process my own grief through drafting this manuscript, and another year later, I returned to it. The writing for this autoethnography began some months before I knew I had advanced stage 1 papillary thyroid cancer in May 2023, and ended a few months after I defended my dissertation and got a divorce finalized in April 2025. Those grieving processes may be topics for the future—especially how I did not actually have any cancer diagnosis at the time when I wrote about speculating that some cancer would hypothetically grow in my throat.

From the excerpts and analyses I choose to share presently, I provide personal and professional perspectives on the concepts of disenfranchised grief, ambiguous loss, anticipatory grief, and secondary loss. After the resurgence of genocide in Palestine began, I added a fifth concept that was not originally a part of the workshop I attended. Collective grief, the final concept I write about, made me realize how much my feelings of hopelessness have resurfaced in the past fifteen months. I identified deeply with Gazan mothers who are survivors of loss, even meeting a mother and her daughter in the hospital locally and bearing witness to their lived experiences. Yet, because I know from research what tools are necessary to combat these feelings of grief, I also realize my immense gratitude. Practicing gratitude is an evidence-based tool and a faith-based tool that anyone can implement.

Finally, in the discussion of this paper, I delineate collaborative autoethnographic methods for community-based participatory research. Although my ideas may not apply to some research approaches or communities, I do anticipate an etic relevance, i.e., ecological validity, in sharing my lived experience. Whether it is simply the fact that grief is a universal lived experience, or that my social identities resonate with certain individuals or groups, my aim is to promote a more interpretivist and constructivist, rather than positivist or objectivist, manner of conducting suicide research with majority-world communities like Muslims, as researchers have done in the past (Lenette and Boddy 2013; Lenette et al. 2013; Kasherwa et al. 2024; Lenette 2022, 2023).

### 1.4. On Suicide, Suicidality, and Violence: Terminology Matters

I take an epistemological and ontological understanding of suicide as self-targeted violence, resulting from the internalization of historical, institutional, and interpersonal violence that is reflected at the intrapersonal level. In my view, the distinctions between suicidality, suicide, and violence are simply differences in the intent of violence—whether it results in self-harm or injury, self-inflicted death, or attempts to kill entire populations through abusive systems of power, respectively. Hence, in my academic view, I prefer to define these distinctions by the use of the words “suicidality,” “suicide,” and “violence.” Through my stance, I assert that suicidality refers to internalized violence that results in nonfatal ideation and attempts, suicide refers to internalized violence that results in self-targeted fatal attempts, and violence itself refers to the fatal intent of abusive systems of power toward minoritized social groups. This stance is supported by scholars such as Alvarez et al. that describe mechanisms for racism as the internalization of systemic violence for ethnoracially marginalized groups (Alvarez et al. 2022). Other scholars have demonstrated strong links between intimate partner violence and suicidality after systematically analyzing past research studies on topics like gaslighting (Sweet 2019; Decker et al. 2018; Campbell et al. 2021; Cavanaugh et al. 2011). Furthermore, the idea of suicide as internalized violence or internalized aggression is not new and emerged as early as 1938 with Karl Menninger, who authored the widely cited Man Against Himself (Menninger 1938).

### 1.5. Theoretical Framework and Conceptual Model

**Figure 1.**
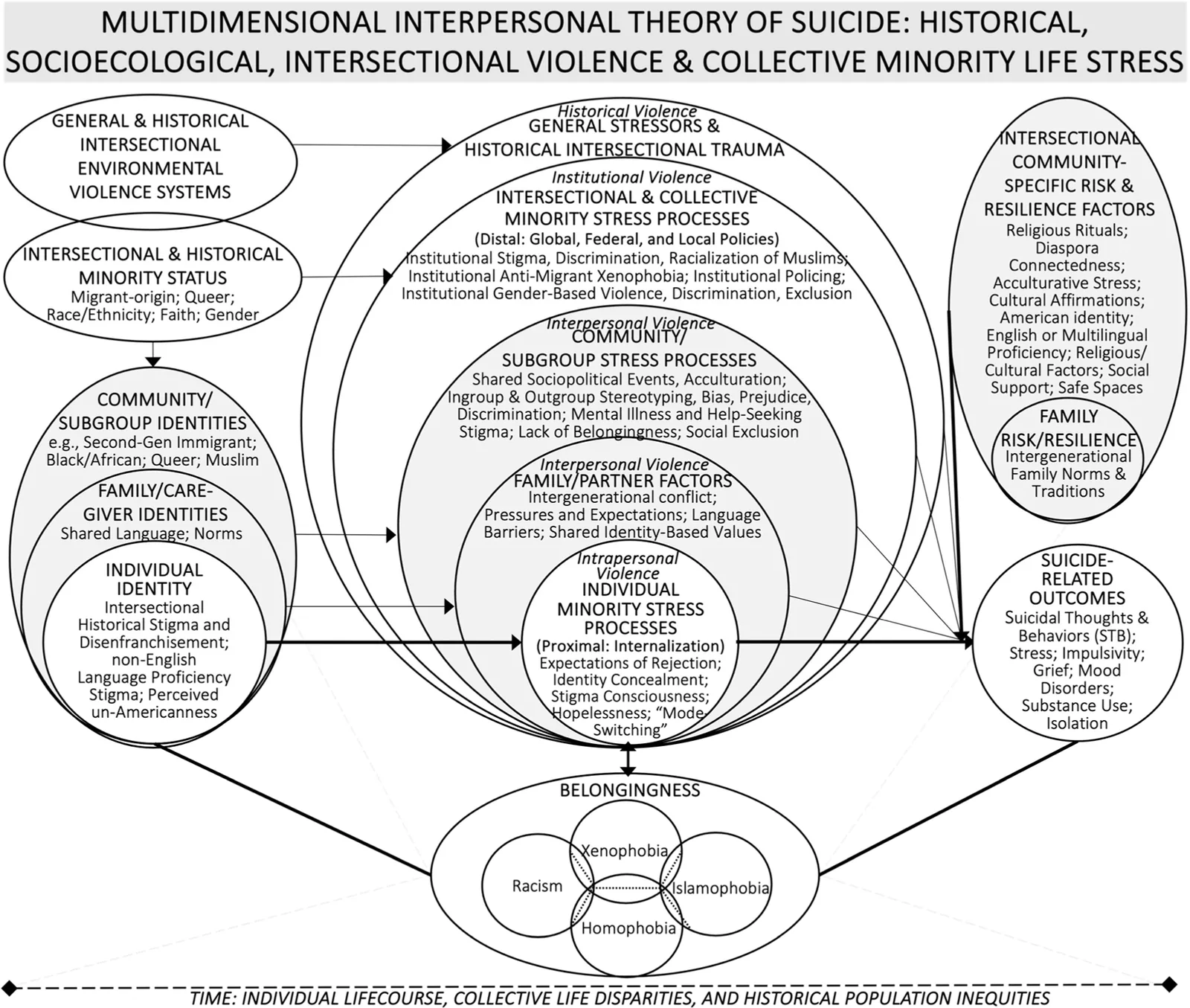
Combining elements of interpersonal theory of suicide, minority stress, intersectionality, historical trauma, and socioecological theory to understand self-targeted violence from a collective life course dimension that perpetuates inequities over time.

Lived-experience-led research disrupts traditional academic literature that perpetuates epistemic violence in studying suicide or other topics from positivist logic (Higgins and Lenette 2024). For the purpose of offering a merged academic and lived experience lens to the study of suicide, I developed a conceptual model in Figure 1 that depicts theoretical frameworks through a “Multidimensional Interpersonal Theory of Suicide: Historical, Socioecological, Intersectional Violence & Collective Minority Life Stress.” This framework visually organizes the multi-layered violence systems that shape individual suicidal ideation and attempts to multiply marginalized individuals under the broader understanding of community or collective stress over the life course. It expands on Joiner’s interpersonal theory of suicide by integrating historical trauma theory inspired by Sotero (2006), postcolonial theory inspired by Said, Meyer’s minority stress theory, Crenshaw’s intersectionality theory, Bronfenbrenner’s socioecological systems theory, Hatzenbeuheler’s theory of stigma as a fundamental cause of health inequities, Fords’ critical race health praxis, and Elder’s life course theory, offering a complex diagram of suicide risk and resilience as both a process and product of converging historical, institutional, interpersonal, and intrapersonal violence (Ribeiro and Joiner 2009; Sotero 2006; Said 2016; Meyer 1995; Crenshaw 2013; Vélez-Agosto et al. 2017; Hatzenbuehler 2016; Ford and Airhihenbuwa 2010). The grey-colored circles represent an emphasis on interpersonal violence and stress processes as critical elements to suicide-related outcomes, expanding on prior theoretical contributions to explain my embodied life story as a Muslim within the global diaspora.

Read from left to right, the left section of the model informs the central core of concentric circles that reflect violence as a multidimensional phenomenon. At the right section of the model appear the community-specific risk and resilience factors, which include a nested circle of family resilience factors. Suicide-related outcomes are at the end of the model, shaped not only by socioecological dimensions but also by dimensions of time. Toward the bottom of the model, there is a closer inspection of the concept of belongingness, which is heavily emphasized in the interpersonal theory of suicide; in my version of this theory, an intersectional dimension of belongingness is important to consider not only for the individual life course but also the collective life course of the communities and populations in which such individuals are grouped socially. Importantly, belongingness is fluid over time and challenges with belongingness must be understood as a dynamic process in which cultural hegemony and norms shift within different contexts. In this way, time is racialized in the life course, as suggested by public health scholars Gilbert Gee and David Williams (Gee et al. 2019). Thus, the life course perspective demonstrates how suicide-related outcomes such as stress, grief, and isolation are contributing factors to the overall historical and persistent inequities in life expectancy for minoritized peoples. In essence, my conceptual model explicates mechanisms of suicide risk building on previous concepts like the weathering hypothesis (Geronimus et al. 2006) and allostatic load (Thomas 2006), racial battle fatigue in higher education settings (Fasching-Varner et al. 2014), and the biopsychosocial model of racism (Clark et al. 1999).

Through this model, I find a way to make sense of my lived experiences as a socially biracial Muslim, non-binary genderfluid woman, with a bilingual diasporic connection to Arabic speakers in conjunction with a postpartum bipolar disorder. The central portion of the model situates these identities within nested systems of community, family/partner, and individual stress. These levels mirror socioecological theory by highlighting how broader systems—sociopolitical events, discrimination, mental health stigma, language barriers, and familial pressures—cascade into more intimate realms of interpersonal (partner, family, and community) and intrapersonal (self-targeted) violence. It is important to note here that while I have not shared any details of my experiences with intimate partner violence nor details of my disability experiences as a chronically ill cancer survivor with Postural Orthostatic Tachycardia Syndrome (POTS) in this autoethnography, I do believe that the model offers a comprehensive emic and etic perspective that can contextualize these embodied experiences broadly. For example, at the individual level, minority stress theory (Meyer), and life course theory (Elder) help frame how cumulative stressors—identity concealment, “mode-switching,” stigma consciousness, and hopelessness—are internalized over time. These chronic exposures to marginalization align with my own lived experience in navigating expectations of rejection in white-dominant, cisnormative, and Islamophobic spaces. The model aligns with the critical race health praxis which demands that such lived experiences testify to embodied narratives of structural racism and settler-colonialism.

The right side of the model centers resilience and risk as community- and familymediated phenomena. It acknowledges protective factors such as diasporic connectedness, multilingualism, religious rituals, cultural affirmations, and safe spaces. These are crucial sources of collective resistance and postcolonial survivance, especially in the context of spiritual and cultural healing. However, the model also points to the tension between resilience and grief, reflecting the ambiguous loss, anticipatory grief, disenfranchised grief, and secondary losses that the author experiences both personally and collectively—particularly in relation to the genocide and suicide in Gaza, Palestine, and the intergenerational silencing of Arabic language. Finally, the outcome domain includes suicidality, mood disorders, isolation, and substance use—common endpoints of a complex system of oppression. Yet this model resists individualizing blame, emphasizing instead that suicide is a deeply social, cultural, and historical phenomenon (Kral 1998; White et al. 2015). In line with stigma as a fundamental cause, suicidality is not merely a symptom of individual pathology but an embodied consequence of systemic exclusion and dehumanization.

Therefore, by grounding the model in lived experience through autoethnography, I embrace an emic insider participant–researcher positionality, offering a methodological and conceptual approach that centers epistemic justice. This work invites suicide researchers—particularly those working with minoritized or majority-world populations—to embrace critical, grief-informed, decolonial approaches that honor cultural specificity and personal voice as opportunities for scholarly insight and political resistance, in line with previous works featured in the book *Critical Suicidology: Transforming Suicide Research and Prevention for the 21st Century* by editors White et al. (2015). Relatedly, this model promotes not just a framework for better understanding how to prevent suicide, but it also posits a more comprehensive map for anti-violence intervention.

In so doing, the future of anti-violence research and intervention should center not just lived experiences but also indigenous methodologies that include spirituality in the conceptual model. Thus, on this final note, I must briefly comment that, in my view, the suicide-related outcome of isolation is one that is consistent with Islamic indigenous perspectives that argue isolation as a spiritual mechanism for satanic possession. This mechanism is described in the saying, or hadith, of Prophet Muhammad, which has been authenticated: “Indeed, the wolf devours the lone sheep who separates from the flock.” This saying can be loosely interpreted to mean that deviating from congregational worship practices and social ties to the Muslim community, or ummah, leads to disconnection from the Islamic lifestyle and thus to isolation, which functions as a welcome entry for Satan to occupy the embodied spirit. While this spiritual mechanism is not depicted in the conceptual model, there is an existing model explicating the concept of suicide in Islam by well-known Islamic scholar, psychiatrist, and researcher Rania Awaad. Awaad’s model integrates disciplines of sociology, psychology, anthropology, developmental science, neurotheology, and Islamic jurisprudence to better understand suicide risk.

### 1.6. Positionality Statement

I bear witness that God, Allah, is one and I bear witness that the messenger of God is Prophet Muhammad (peace unto him).

**Figure 2.**
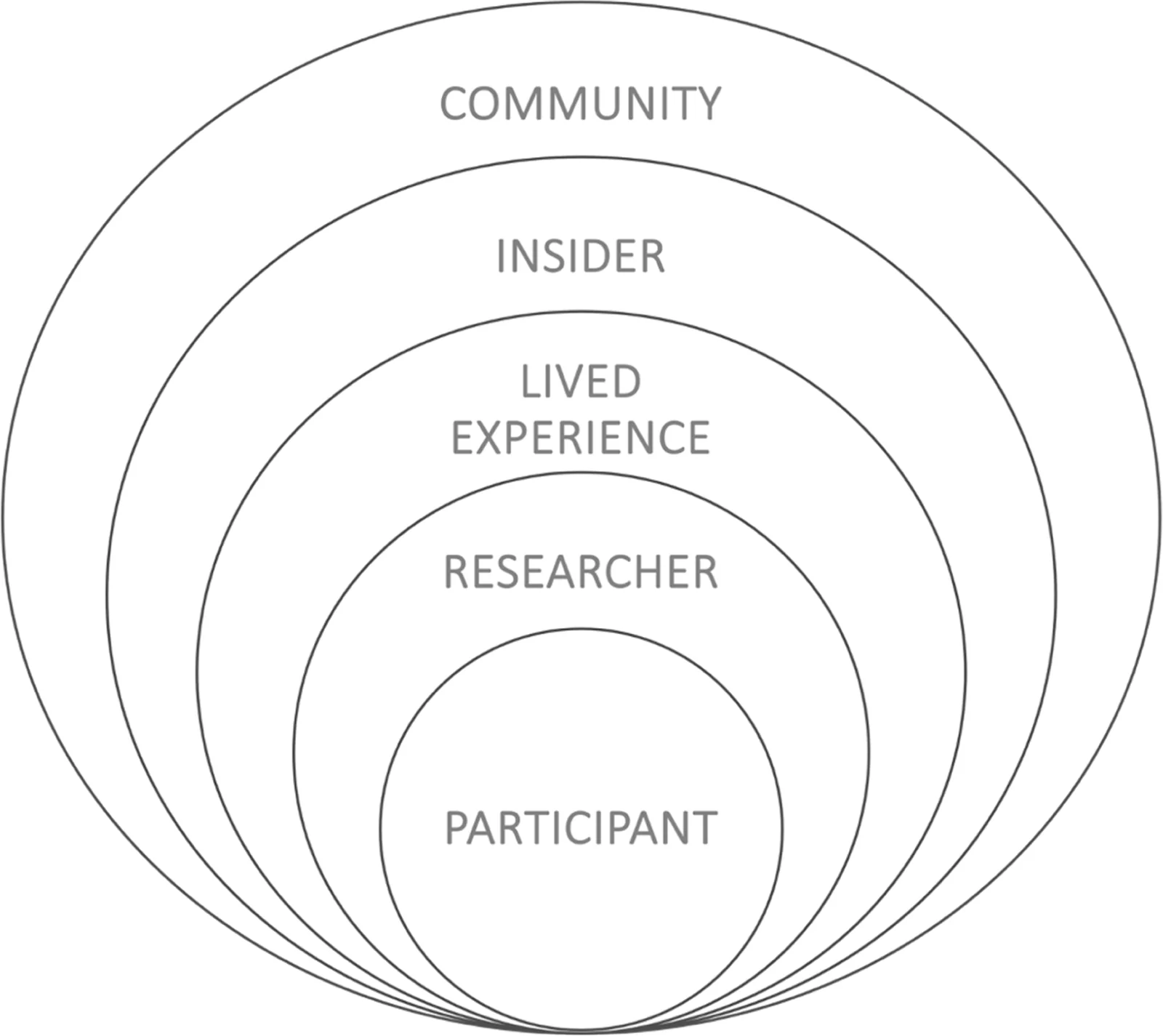
Emic positionality depicted in concentric circles.

First and foremost, this declaration of faith—or *shahada*—is the most critical aspect of my positionality as an insider researcher. I also bear witness to the pain, suffering, pleasure, and joy of people who identify as Muslim in the global diaspora. I also bear witness to residing on occupied lands that were historically stewarded by the ancestral tribes of the Piscataway peoples in what is known as Baltimore, Maryland, United States of America. While I am recognized as a U.S. citizen, my strong preference is to identify first and foremost as a member of the global Muslim diaspora. I was born and raised Muslim in the ancestral lands of the Tongva people, also known as Southern California. I am the third daughter and last child of immigrant parents who identify as Pakistani and were born on the Indian subcontinent. As demonstrated in Figure 2, my work is oriented from an insider perspective, allowing me to hold presence in the U.S. Muslim community as both a participant and researcher simultaneously. Because I am a survivor of suicide attempts, it is critical that I both acknowledge the role of my lived experience and continue to advocate for violence prevention as a part of my joint personal and professional values of social justice.

As I identify as a survivor of suicide attempt, I am casting my lived experience as a key tenet to the study of suicide prevention and anti-violence research. Thus, as a Muslim social and behavioral scientist conducting community-based participatory research, my positionality is as an insider participant–researcher. In contrast to solely being an insider researcher, as a participant, I acknowledge that I am participating with the community itself in receiving services, engaging in dialogue, and ultimately shaping advocacy for preventing suicide. Being a participant allows me to share multiple dimensions across place, time, language, social circumstances, social intimacy, and lived experience with others in my community that, even among other insider researchers, is not cultivated (Ngunjiri et al. 2010; Kaniuka and Bowling 2021; Kuper et al. 2018). Importantly, in this work, I refer to the concept of “research” as a scientific practice, and the term “scientific” to generally refer to any methods developed for social studies. As demonstrated by the diagram of concentric circles, I self-identify as a member of the global Muslim community, and such, I hold insider status within the United States context. Additionally, this *emic* perspective as an insider is enhanced by my personal lived experience as a suicide attempt survivor, which I critically analyze in this autoethnography from the perspective of a researcher who has acted as a ‘participant’ within the community, including at events, programs, and conversations locally and nationally.

## 2. On Identifying Multidimensionally as “Bi-___” as Analyzed Through Disenfranchised Grief

### 2.1. Excerpt: Bi^1^

By appearance and perception, it is not clear “what” I am, let alone “who” to the Orientalist eye. One time, I was asked “Where are you from?” by a white man in Huntington Beach, California. I countered, “outer space.” What was once a deeply loaded question eventually led me to a lifelong journey of healing from violence, including suicide.

Where do I go to feel seen? I was born and raised in sunny Southern California as a second-generation immigrant. My parents come from Bihar, India. Should I act Pakistani, Muslim, brown, American—full-time—or just channel whatever frequency occurs momentarily?

Could I share common feelings with people who identify as biracial? Which societies would outcast me if I came out as a genderfluid non-binary woman? What makes disclosing bipolar depression a question of morality and ethics: to institutions, colleagues, friends, human subjects?

Where is my camouflage today, do I modify it to ‘pass’ or adapt? How will I know my identity is celebrated, not stigmatized, across time and dynamic social networks? Is my lived experience the embodiment of stigma as a fundamental cause of health inequity?

(Hatzenbuehler et al. 2013)

Can empirical evidence attest I am not the only one feeling this way? Would my faith community support my scientific views? Would the scientific community support my spiritual views? Who can I trust? How could personal narratives sustain and promote empowered lives in diverse populations?

Why does such a great task befall me? My life mission…to reclaim humanity? I am disabled; I need more spoons.^1^ Living means so much more. Should I hope for a better afterlife? Will my hope run out? End this isolation, I can’t take it anymore! I pray for peace in all souls.

### 2.2. Bi^1^ Analysis: Disenfranchised Grief

I describe my life through a broad lens of disenfranchised grief in the introductory excerpt entitled “Bi^4^.” Referring to any grief that goes unacknowledged or unvalidated by social norms, disenfranchised grief is also known as hidden grief or sorrow (Muslim Mental Health Conference 2022). In disenfranchised grief, the rules or norms that society assigns to grieving, in essence, are not neatly codified, giving the term a certain breadth and flexibility in its application to a wide range of feelings concerning grief that may be difficult to understand (Doka 1999; Corr 1998). In other words, “there is no social recognition that the person has a right to grieve or a claim for social sympathy or support” (Doka 2014). Beginning with my first thought of “it is not clear ‘what’ I am, let alone ‘who’ to the Orientalist eye,” I suggest a displacement between my own sense of self and the loss occurring due to how others perceive this sense of self. I seem to struggle with being simultaneously visible yet invisible. In my use of the words “by appearance and perception,” I indicate some metacognition of my place in society, noting that these factors place me as a subject bound to be interpreted differently in various contexts, though notably the primary focus is through a colonial perspective with reference to Orientalism. Orientalism has been critiqued as a reductionist representation of Asia and the Middle East, with stereotypes of Orientalists embodying colonialist attitudes. Within this context, my hidden grief appears to be a result of incongruence between my identity and environment.

In the following sentence, it is made clearer who is objectifying me as a subject, referring to a white man from Huntington Beach, California, a predominantly white, upper-class, and conservative city. Based purely on my foreign-appearing nature, I sardonically describe myself as being from “outer space,” later sharing this feeling of otherness with use of the phrase “embodiment of stigma.” Carrying on with the feeling of otherness, I ask, “where is my camouflage today?” and reflexively inquire, “do I modify it to ‘pass’ or adapt?” It is unknown whether I am asking the reader or speaking to a larger and more abstract audience in a rhetorical sense, suggesting some tension between anonymity and acceptance. This covert communication style is furthered with my questioning: “Can empirical evidence attest I am not the only one feeling this way?” Suggesting that there might be evidence basis for validating my sense of self, or who it is I am, I speak in a tone of abject hope. Further indicating a deep sorrow, I ask, “Why does such a great task befall me?” as if to existentially question my motivations to live. These inclinations are grounded in the fact that I disclose my “lifelong journey of healing from violence, including suicide.” Research definitions drawn from the United States Center for Disease Control on suicide categorize this health outcome as an act of self-directed violence or injury with intent to die; and, some suicide researchers place suicide under the broader umbrella of structural violence resulting from intersecting systems of oppression and institutionalized stigma, e.g., racism, sexism, Islamophobia, heteronormativity, homophobia, or nativism (Alvarez et al. 2022; Samari 2016; Perez-Brumer et al. 2015).

A characteristic of disenfranchised grief is that it is “grief that persons experience when they incur a loss that is or cannot be openly acknowledged, socially sanctioned, or publicly mourned.” I convey such grieving by asking, “Where to do I go to feel seen?” Because others often minimize or do not understand disenfranchised grief, it is a form of grief that I find particularly hard to process and work through. By asking myself about whether I share “common feelings” with others who identify as biracial, and which societies would “outcast” me for coming out as genderfluid non-binary, and questioning “morality and ethics” for disclosing my bipolar depression, I outline three dimensions of identity that intersect. Through the title of “Bi^5^”, I imply that these dimensions are exponentiating, or a product of the intersections they multiply. The “bi” dimensions that I identify are biracial, non-binary, bipolar, and bilingual. These identities are stigmatized in mainstream American society, inflicting disenfranchised grief within me, leading me to ask, “How will I know my identity is celebrated, not stigmatized, across time and dynamic social networks?” Furthermore, I wonder whether my lived experience attests to past theories relating to stigmatized identity, namely what is known as the fundamental cause of health inequity that states people from stigmatized groups face suicide disparately due to being an outgroup (Hatzenbuehler et al. 2013).

Finally, the concept of “self-disenfranchised grief” is apparent in the final two paragraphs with a common theme of questioning whether the loss I experience is even worthy of mourning or bereavement. Loss, in this sense, is my questioning of whether “hope will run out” and that I am “disabled” and “need more spoons”—a phrase commonly used by chronically ill or disabled people to describe finite energy to carry out tasks on any given day, typically measured on a scale of ten spoons. By asking “should I hope for a better after life?” I suggest that my life is not worthy of living, perhaps to intimate the larger worthiness of my grieving. Again, since it is unclear who I am speaking to, I am attempting to assert the worthiness of my grief by writing about it and allowing it to hold space and be seen or read. It is possible that my writing may never be read—or, if read, misunderstood—adding evidence to my feelings of being unseen, or unworthiness of existing, and overall, my feelings of unworthiness toward life. “Living means so much more,” I assert. Hope seems to centrally connect with the idea of worthy grief and worthy life, when I speculate on whether my “hope will run out.” As key factors in suicide research, hope and hopelessness are predictors of suicide, as is isolation (Polanco-Roman and Miranda 2013; Ribeiro et al. 2018). By connecting hope and isolation, I associate these two concepts before concluding with statements of pain and prayer: “I can’t take it anymore! I pray for peace in all souls.” In my final sentences, I reflect on the worthiness of my life and—in corollary—the worthiness of my bereavement, being found through faith.

## 3. On Identifying Beyond the Binary as Analyzed Through Ambiguous Loss

### 3.1. Excerpt: Bi^2^ as Genderfluid Non-Binary

2008 would be the birth year for some early adults who are age 15 today. That was a monumental year for so many reasons. As was customary in my older sister’s household, we discussed most matters openly. California’s Prop 8 was distressingly instigated. “I am not a member of that community, so I didn’t vote on it,” I gently protested. I felt courage to speak up on the matter but suppressed my pain simultaneously. It happened to be the first year I was eligible to vote as I had just turned 18.

Why do I feel such injustice when my loved ones speak against civil rights for queer people? I just know what it feels like to experience crippling discrimination and I relate to having no idea who or what is safe to ‘be’ around. Now, in 2023, the conversation has evolved, as have I. Ironically, Muslim elders and Islamic teachings led to me embracing my queer vibes. During a session with my spouse around 2019, our Black Muslim marital therapist conveyed she sensed a strong masculine energy from me. I was taken aback.

Last year, I encountered messaging on Instagram from a nonprofit organization called HEART. It talked about two of the 99 names of Allah. Names describing God as “compassionate” derive from the Arabic term for “womb.” In fact, it is a divine characteristic of God to be outside social constructs such as gender. He is not “he.” Is this affirmation what I had been looking for all along? Finally, I get it, I am enby, genderfluid, too, as a parent, a mom. Many creations gender bend for survival. The Creator divinely fashioned me.

God makes no mistakes. God says “Be! and it is.” So, I am.

### 3.2. Analysis: Ambiguous Loss

Ambiguous loss occurs when grieving someone who is still alive or when someone or something profoundly changes or disappears from what existed before. In this passage entitled “Genderfluid Non-Binary,” I indicate a transition between a conforming gender identity such as female and a non-conforming one, i.e., “genderfluid” and “enby” (non-binary), stating that I have “evolved” to “embracing my queer vibes.” Genderfluidity is recognized as rejecting the rigidity of a fixed gender identity (Galupo et al. 2017; Diamond 2020). The context of the ambiguous loss is situated within me as an individual, though it is arguable that the loss occurs within my family and community systems, as I do mention these as a part of the larger interconnected context and setting within which they exist: “I am.” In proclaiming my newly gendered existence, I offer a multi-layered understanding of myself. I cite that “God makes no mistakes,” using a phrase from the Islamic holy book, the Quran, which states that God says, “Be! and it is” (Quran 3:47). In parallel with the earlier analysis of disenfranchised grief in which I signify the worthiness of my life, it is possible that my ambiguous loss questions whether a profound change in identity makes my life more worthy of living. As if to implicitly respond to this question, I bring up the topic of “affirmations,” implying that statements like “Many creations gender bend for survival,” and “The Creator divinely fashioned me,” remind me that my life is worthy of living. Extending this, I may be realizing that my profound change in gender identity is both a part of my self-disenfranchised grief and a part of the existential nature of my suicidality.

Through my outlining of a timeline or process leading up to this ambiguous loss—i.e., identifying as a genderfluid non-binary woman (which falls under the umbrella of transgender)—I bring up themes around family, community, safety, and injustice. As if to allude that each of these themes are inherently bound together, I cite “crippling discrimination” and “civil rights” as central to my ideas of justice in the same paragraph I describe feeling “pain” and “distress” around “loved ones” such as family and community who “instigate” conversations pertaining to queer people, presumably with a negative connotation. Previous research on the topic of ambiguous loss and trans-identities exists, particularly in the context of family, which is critical to bring a greater awareness of my own experiences with my own family (Wahlig 2015; Norwood 2013). In a publication entitled “Grieving Gender: Trans-identities, Transition, and Ambiguous Loss,” author E. Norwood describes that “family members experience transition as a living death, wherein the trans-identified person is perceived as somehow present and absent, the same and different, at once” (Norwood 2013). Norwood centers the family’s meaning-making of trans-identity as a core tenet of enduring and overcoming ambiguous loss “as a living death,” further explicating the following:

Even for those trans persons who do not transition from one sex category to another, moving to a more gender-fluid identity involves a shift away from one if not clearly toward another. For family members, transition brings about a renegotiation of meaning regarding the trans person’s identity, which often incites ambiguous loss. This loss is related to contradiction in the meaning making process resulting in the perception that the trans person is both present and absent, the same and different.

(Norwood 2013)

In relation to the passage, I center “Muslim elders and Islamic teachings” as the topic of the second paragraph as if to implicitly demonstrate some inherent contradiction between my gender identity and my Islamic background and involvement with the Muslim community. Offering memories from my marital therapy sessions and messaging from social media, I extend similarities between my own gender identity and the socially assigned gender of God, noting that it is a divine quality to be gender non-conforming, as God is beyond socially constructed norms of gender. Using one of the Islamic 99 names of Allah—”the Creator” (*Al Khaliq*)—to validate and affirm myself, I again draw from my faith tradition to “get it” or make sense of who it is I am—that I am “divinely fashioned” and that this fact should be recognized as a cause for living, and for not desiring death nor wanting to kill myself. As other Muslims attest, such as in the book *Hjiab Butch Blues* by Lamya H, the spiritual struggles of queer identity exist as tensions across diaspora communities worldwide (Rouhani 2017; Yip and Khalid 2012).

### 3.3. On Gender and Suicide

It is important to note that transgender people are at a greater risk of dying by suicide than any other sexual or gender minority due to structural violence against non-conforming gender identity, gender performance, and overall gender norms (Duncan and Hatzenbuehler 2014; Miller and Grollman 2015; Perez-Brumer et al. 2015). Transgender suicide rates are high globally and domestically, with some estimates hovering around 30–50% in both contexts (Virupaksha et al. 2016). Suicidal ideation—thoughts of someone considering to kill themselves—is even more prevalent, with some epidemiological estimates exceeding 50% of all transgender and non-binary youth in U.S. domestic contexts (The Trevor Project 2022; Levin 2022; Toomey et al. 2018). Previous researchers have identified community-level interventions as a key level of intervention for people who identify as transgender, using a socioecological approach (Kaniuka and Bowling 2021; Kuper et al. 2018). Gender-based violence also holds strong associations with intimate partner violence (IPV), with some scholars commenting on the sociology of gaslighting and implications for suicidality in people who were targets of IPV (Decker et al. 2018; Cavanaugh et al. 2011; Sweet 2019; Crenshaw 2013).

## 4. On Identifying as a Patient with a Post-Partum Bipolar Diagnosis as Analyzed Through Anticipatory Grief

### 4.1. Excerpt: Bi^3^ as Bipolar

What use is my lived experience to a psychiatrist? As a young person, I felt gaslighted when I would bring up issues that affect my daily life to clinicians. It could be their unintended ignorance, or bias. I spent so many years perseverating, feeling suicidal ruminating over how powerless I felt. Will I grow cancer in my throat because I have grown accustomed to self-censorship for my own survival? When I do speak up, why does it sound like I’m the crazy one?

How do I know whether your diagnosis of me pathologizes discrimination? Are you sure it wasn’t racism-based traumatic stress that twisted the mind of my father, Mohammad, into schizophrenia? Is it adjustment disorder still adjusting since his arrival in the 80s? How do I know I can trust you when diagnostic classifications derive from ungeneralizable samples who do not represent me or my life?

Will my voice count to the medical establishment if I speak in scientific terms? I reclaim power with conviction and diction to describe paranoia or hypervigilance as normal reactions to surviving historically oppressive systems. My world-renowned advisor stated that women of color are disproportionately diagnosed as bipolar. Most psychiatrists and psychologists cannot relate to my disabled, nonconforming, racialized lived experience. At 29 years old, I roll into the hospital of my home institution—the finest in the world—a pregnant patient in a wheelchair. A white nurse speaks to me in Spanish, racializing me as the kind of brown I am not.

I catch myself feeling triggered. I realize I don’t need to be triggered, but now our dynamic becomes racialized. Postpartum, I go back for a routine 6-week checkup. It’s July 2020. I inform the midwife I am symptomatic of psychosis. What? “Psychosis.” She gaped into my eyes, unaware of protocol. Later, policemen show up and forcefully take me away to the hospital by court order. It’s a ‘voluntary’ hospitalization, they tell me to say…

### 4.2. Analysis: Anticipatory Grief

Defined as grief that occurs before a potential loss, anticipatory grief encompasses circumstances that lead a person to think that death or an ending is a real possibility. In the context of living as a person diagnosed with postpartum bipolar, anticipatory grief may be understood as a prolonged process of anticipating one killing themselves, not knowing if it might happen. The possibility of death being self-inflicted, or ideation and behaviors indicating suicidality, is a key characteristic defining bipolar disease. Describing this phenomenon, I mention how much time, in “years,” I spent “perseverating, feeling suicidal ruminating over how powerless I felt.” Questioning my faith in the medical establishment, I begin the passage by positing, “What use is my lived experience to a psychiatrist?” and I lead up to the end of the passage with a story on my eventual hospitalization, with particular emphasis on the word “voluntary.” Using the term “voluntary hospitalization” hints at historical power dynamics of patients with mental illness and the patient-to-prison pipeline (Onah 2018).

Reclaiming my “disabled, racialized lived experience,” I impart a strong tone of injustice about the incongruence between the provider’s positionality and my lived experience as a patient, calling out a lack of reflexivity evident in providers’ “unintended ignorance or bias.” This dissonance contributes to my overarching fears relating to loss of power. I assume that providers are unqualified: “How do I know whether your diagnosis of me pathologizes discrimination?” Given this assumption, it is reasonable that I experience anticipatory grief in imagining myself dying not by direct self-inflicted suicide but indirectly by the forces of the system, e.g., law enforcement or ‘suicide by cop,’ if I fail to comply with the racist medical system.

Anticipatory grief may also build across lifetime experiences such that it becomes a natural coping mechanism to prepare for loss ahead of it actually occurring. In line with this long-term pace, I state that I have “grown accustomed” to self-censorship for my survival, wondering when to speak up, and also providing personal background vis-à-vis disclosure of my father’s schizophrenia diagnosis. Intrinsically enjoined with anticipatory grief is disenfranchised grief for my own family and personal history with mental illness. I state, “Is it adjustment disorder still adjusting since his arrival in the 80s?” unraveling a complex mixture between my racialized and disabled health profile and that of my father’s. My social positioning as a “second-generation immigrant” deliberately frames my anticipatory behavior: whether to act “Pakistani, Muslim, brown, American—full-time—or just channel whatever frequency occurs momentarily?” as it reflects a sort of code-switching that involves switching into different frequencies, or ‘modes,’ of behavior, such as mode-switching into Pakistani versus American as mutually exclusive. Thus, ‘mode-switching’ suggests nuances in distinguishing this type of anticipatory grief as better characterized by “racism-based traumatic stress” versus a clinical label of “adjustment disorder.”

An additional layer contributing to my disenfranchised–anticipatory grief synergy is my experience from a research perspective: “How do I know I can trust you when diagnostic classifications derive from ungeneralizable samples who do not represent me or my life?” This suggests that I am making sense of my experiences not only through the lens of a patient, but also a human, a child, a pregnant person, a researcher, and—as emphatically noted—a member of several intersectionally subordinated and stigmatized social classes. I channel the grief and pain of these silenced voices: “When I do speak up, why does it sound like I’m the crazy one?” and I also question, “Will my voice count to the medical establishment if I speak in scientific terms?” signifying that holding status as a researcher empowers me as it is a means to amplify my voice.

Further making sense of my positionality, I state, “I reclaim power with conviction and diction to describe paranoia or hypervigilance as normal reactions to surviving historically oppressive systems.” In severe mental illnesses such as bipolar, clinicians often search for signs of unusual or abnormal heightened awareness during patient intake. Paranoia has been noted to be characteristic of some forms of schizophrenia, which is on the extreme end of the bipolar spectrum. Because one of the risk factors of developing schizophrenia is exposure to poverty in early childhood, it makes sense that I contest pathologizing their mental illness and instead refer to upstream structural forces, as if to offer psychiatrists’ discerning before diagnosis.

### 4.3. On Mental Illness and Suicide

People with bipolar or schizophrenia are significantly more likely to take their lives compared to other mental illness diagnoses. The life expectancy of people with bipolar or schizophrenia is anywhere between 10 and 18 years less, with gender differences in lifespan (Laursen 2011). One systematic review found a life expectancy of about 67 years for people with bipolar (Chan et al. 2022). Suicidality is nearly 10–30 times higher in people with bipolar compared to the general population, accounting for some of this increased mortality (Dome et al. 2019). Ranked as a top ten leading cause of disability worldwide by the World Health Organization, bipolar I and II hold a combined prevalence of around 1% globally. With bipolar holding such a profile of high morbidity and mortality, I argue that perhaps suicidal thoughts and attempts are embodiments of anticipatory grief. In my final statements, I express how the clinician was “unaware of protocol” and policemen “forcefully” take me to the hospital, insinuating further the anticipatory grief of not only holding a postpartum bipolar diagnosis, but also the anticipatory grief of not knowing whether I would remain alive if committed into the system.

## 5. On Identifying with Biracial Dualities as Analyzed Through Secondary Loss

### 5.1. Excerpt: Bi^4^ as Biracial

Race is socially constructed, right? That’s why Muslims are racialized despite being a faith group. My adolescent years were spent at the Islamic Center of Irvine, where undercover FBI Agent Craig Monteilh infiltrated our Southern California mosque, posing as a new convert, unlawfully targeted our community, abused our sacred spaces, and violated us. (Rafei 2021) We are so often surveilled by undercover agents that I was abnormally distrustful of my own ex-spouse before marrying; he identifies as a convert.

Who counts as Muslim? The answer varies depending on who is asking. FBI? CIA? TSA? Matchmaking aunties? Remarkably, even Muslims don’t agree on who is Muslim. I feel intuitively connected to racialized dualisms of belongingness. I am an insider as a Muslim yet an outsider as American. Or is it the other way around? I could be President, but I am not American enough. Even my own South Asian family ridiculed me: “American Born Confused Desi (ABCD).”

On 9/11 one year, I wrote on Facebook that I would truly “never forget.” The media hijacks my narrative with wrongful Islamophobic messaging about my people. How could I ever have the audacity to call myself a term that does not even belong to me? America spans Canada, America spans Mexico. Sure, I am American, but I fail to be a patriot. Does being American mean I can travel to Israel with a hijab, brown skin, and a U.S. passport without being detained? Am I American then, too, or just racially Muslim?

Muslims who dichotomize hijab—wear it or not—might not consider me as one of their own. How do I conduct community based participatory research if I am not accepted by my own people? Should I just abandon my hypothesis that living as American Muslim is theoretically a biracial or multiracial experience, which scientifically contributes to suicide risk? Who will believe me, and accept my story in science, in community, in media?

### 5.2. Analysis: Secondary Loss

Following my assertion that race is socially constructed (Garner and Selod 2015), I describe themes of grief that span secondary losses enmeshed with disenfranchised grief and ambiguous loss related to a racialized identity (Doka 1999). Secondary losses are usually non-death losses, such as losses related to financial stability, losing a sense of meaning in self or purpose, loss of dreams for the future, and loss of identity that occur over time because of some death (Doka 1999). In this excerpt, this death is assumed to be the ambiguous loss of my visibly Muslim identity, such as discontinuing my practice of wearing hijab. Losses in social support and social status are also a part of secondary loss, and in this case, these would be my lost sense of self and place in society because of my Muslim identity being targeted. Like it was for my disenfranchised grief, I struggle to make meaning of “who is Muslim,” citing also that I am “not American enough” and “ABCD,” which means “American Born Confused Desi,” whereby Desi is a person of South Asian heritage.

Centering a narrative about 9/11, I allude to social stigmatization by mentioning the popularized patriotic phrase of “never forget” in contrast to “my people,” supposing a duality of mutual exclusion between being American and being Muslim, respectively. A version of double consciousness (Black 2007), the complicated interplay of negotiating between these two identities constitutes an ambiguous loss as also a disenfranchised grief. This also results in both the loss of my identity and my sense of self as chief secondary losses. Secondary losses, in this sense, are common to the multiracial lived experience. People who identify as multiracial, multicultural, or multiethnic, especially in young age, experience higher rates of suicidal ideation or attempts (Subica and Wu 2018). Some explanations for such suicidality can be understood through the interpersonal theory of suicide, which endorses the critical interpersonal need of belongingness in preventing suicide. Indeed, the aspect of lacking belongingness is a core part of grieving my secondary loss, particularly in my struggle to make meaning of society’s racialization of who I am as a Muslim—though I am not alone in coping with Islamophobia (Kunst et al. 2012).

One aspect of secondary loss that pertains to this passage is my loss of dreams for the future. In stating whether I should “abandon” my hypothesis about my racialized experience, and in asking “who will believe me” and “accept my story,” I indicate a sense of hopelessness. This loss of confidence, loss of faith, and loss of social support—both perceived and actual—are central to secondary loss (Pihkala 2024). These factors also beckon the disenfranchised grief expressed in the first excerpt: “Why does such a great task befall me?” In my existential dread, I suggest a lack of faith in myself, similar to the tone of hopelessness aforementioned. Although not directly stated, I am also subject to the secondary losses of income and financial security that result from the ambiguous loss of my American identity and institutionalized Islamophobic discrimination.

Because employment is a path to long-term sustenance, it is possible I am mourning the future secondary losses of economic stability that result from systemic anti-Muslim racism and Islamophobia. What substantiates this loss is fear rooted in my past experiences, where an undercover agent “infiltrated” my mosque community and performed unlawful surveillance.

### 5.3. On Socioeconomic Status and Suicide

Previous research on health inequities due to Islamophobic racism exists and has direct implications for mental health intervention (Samari et al. 2018; Kunst et al. 2012). Socioeconomic status is a major predictor of suicide, with over three quarters of the world’s suicides occurring in the majority world, particularly in Low to Middle Income Countries (LMICs), such as the ones that are of my ethnic and faith background. Previously, scholars have called for suicide surveillance in LMICs (Vijayakumar and Armstrong 2019); however, I believe there must be special considerations for Muslim-majority states, as surveillance may be conflated with intelligence. Scholars who engage with indigenous communities have demonstrated efficacious interventions in preventing suicide which serve as a source of guidance (Cwik et al. 2019, 2016, 2014). Recently, a series of academic articles commissioned by *The Lancet* reveals more in-depth knowledge on the global epidemiology and etiology of suicide (Moran et al. 2024).

## 6. On Identifying with Arabic-Speaking Bilingual Diasporas as Analyzed Through Collective Grief

### 6.1. Excerpt: Bi^5^ as Bilingual

Arabic script on an envelope of a letter I wrote to my advisor read: “In the Name of Allah, the Most Beneficent, the Most Merciful.” On the evening of October 7, 2023, joined by colleagues and my children, I was enjoying dinner at the home of my advisors. I passed by their kitchen, and saw a little cubby with ornaments and decorations, showcasing this letter I had written years ago. It was not long after that I received news of what happened overseas. God placed me there at that time, so divine. I passionately prayed for my colleagues, including Dr. Bahzad AlAkhras, a child psychiatrist native to Gaza, who I had met through the State Department just five months before.

They say that the genocide began that day, yet I disagree. As a part of the greater Muslim diaspora, or Ummah as we say in Arabic, I knew this was just a resurgence of the violence and forced displacement that Palestinians have faced over the past century. Although I do not fluently speak in Arabic, I knew enough to get by in my travels to Arabic-speaking countries in West Asia and North Africa. I had no consoling words to share with my colleagues in Gaza, when, within a week, I found out that some of them and their families were murdered early in the first days of the war outbreak. What do I say, in Arabic, to my Gazan colleagues overseas, to convey what was in my heart? I did not have the words, not even in English.

The Prophet Muhammad (peace and blessings upon him) stated: “None of you should wish for death because of a calamity befalling him; but if he must wish for death, he should say: ‘Oh Allah, keep me alive so long as life is better for me, and let me die if death is better for me.’” This is a saying, or hadith, I only found recently. Now, it is January 2025, and with the implementation of a ceasefire plan, my only prayer is that the lives of innocent Israelis and Palestinians alike are spared. Why is there no birthright for Palestinians? How can we reconnect with our roots?

Before we prayed in the direction of the Kaaba, or house of God, in Mecca, Saudia Arabia, our Muslim ancestors prayed in the direction of the Al-Aqsa Mosque, also known as the Dome of the Rock. This is where the Prophet Muhammad miraculously ascended to the heavens and directly spoke to Allah on the holy night known as Israa wa Miraj. He met with Prophets Adam, Abraham, Moses, and Jesus. It was the night when our Islamic practice of the five daily prayers were sealed, as the Prophet spoke directly to Allah on ordaining them. When I prayed in Jerusalem at the Dome of the Rock in 2012, in the small cave underneath the prayer area, I felt connected to Allah and to the people of Jerusalem—the Hebrew speakers and Arabic speakers, alike.

We share so many ancestors, yet conflict persists. Though geographically disconnected, every night I went to sleep and every morning I woke up, over the past 15 months, all that connected me to Gazans was an overwhelming survivors’ guilt. Every shower I took, every sip of water I drank, every jacket I wore, every bite of food I ate—all of it was a part of my daily mourning. If I could speak better Arabic, maybe I could process the pain better. But suffering has no language, and neither does sympathy. Our universal language is prayer.

“In the Name of Allah, the Most Beneficent, the Most Merciful.”

### 6.2. Analysis: Collective Grief

Collective grief is defined as a type of public or group mourning to a shared event, such as a natural disaster, war, or other mass tragedy (Køster and Kofod 2023). In the excerpt “Bilingual,” I carefully avoid conflation between the Muslim diaspora and Arab diaspora; however, the collective grief of the Gazan genocide is one that impacts many Muslims worldwide, including me. Though Arabs only comprise about one in every five Muslims globally (Mapping the Global Muslim Population 2009), most practicing Muslims in the world know some level of Arabic to perform prayers, even if Arabic is not an official state language or culturally spoken in Muslim-majority countries. This fosters our sense of collectivism, which is inherently a part of our faith, to understand ourselves in the context of the *ummah*, or global diaspora.

At the beginning and end of the excerpt, I deliberately bookend the Quranic verse “In the Name of Allah, the Most Beneficent, the Most Merciful.” This is the most common verse in the Quran, which appears at the beginning of 113 of the total 114 chapters, and one that Muslims recite in Arabic at least 17 times a day during the five daily prayers. In referencing it, I assert my connection to Islamic faith as a way of overcoming the collective grief that I and so many in the Muslim Ummah are experiencing worldwide through the genocide. I state that I “passionately prayed for my colleagues, including Dr. Bahzad AlAkhras,” (Alakhras 2024), noting that despite our geographical distance, I was grieving losses with him, our shared colleagues, the Gazan and Palestinian children and adults, and Arabic-speakers as well as Muslims across the world, collectively.

As Muslims, we believe that Prophet Muhammad, peace be upon him, instilled this sense of collectivism in stating, “Believers in the *ummah* are like one body; when one part of the body feels pain, the whole body feels pain.” For this reason, I call to question whether the genocide began on 7 October 2023 when stating, “They say that the genocide began that day, yet I disagree.” Instead, I view the genocide as a “resurgence of the violence and forced displacement,” as if the body has been in continual pain without healing. Even though I am not physically in danger as Gazans and Palestinians feel, I share in collective despair. As a non-native speaker of Arabic, I question how best to express this abject feeling in saying, “I had no consoling words to share with my colleagues in Gaza…What do I say, in Arabic, to my Gazan colleagues overseas, to convey what was in my heart? I did not have the words, not even in English.”

When I cite the hadith invoking to “keep me alive so long as life is better for me, and let me die if death is better for me,” I speak to grief from a faith-based lens. I defer to divine wisdom, as we do in Islamic faith, to be certain that God works in mysterious ways, and to view death not as an opportunity to end corporal or psychological suffering. I also question if there are ways to mitigate this suffering by citing the need for “birthright for Palestinians” to “reconnect with our roots,” citing a moment when “I felt connected to Allah and to the people of Jerusalem—the Hebrew speakers and Arabic speakers, alike” when praying at the *Al-Aqsa* Mosque (Golden Dome Mosque). Yet my “overwhelming survivors’ guilt” is apparent in stating the privileges of having water to drink, food to eat, and clothes to stay warm. I feel grateful for my privilege, even as during 6 months of the genocide, I was housing insecure myself.

There is no doubt that I imagine many suicides occurring in Gaza, as suicide is a direct and indirect product of structural colonial violence, i.e., apartheid, as a form of ongoing genocide. In the occupied Palestinian territories, and in the Gaza strip, such genocidal apartheid takes form as linguicide. Genocide tactics have targeted the decline of Arabic-speaking populations of all religious backgrounds for multiple decades. According to the Chair of the Palestinian Ministry of Mental Health, Dr. Samah Jabr, Gazans—who are majority Arabic speakers—face chronic traumatic stress disorder, not post-traumatic stress disorder, or PTSD (McKernan 2024). This type of stress results from ongoing violence exposure, and correspondingly increases suffering and hopelessness (Polanco-Roman and Miranda 2013), which are two of the most pervasive risk markers for suicide across populations. Furthermore, of the 2 million Gazans, more than half are under the age of 20, and are currently facing multiple adverse childhood experiences (Alakhras 2024), yet data collection to prove this is wanting (Jones et al. 2020). The Gazan and Palestinian populations face: military law, systematic starvation, inaccessible clean water, lack of health infrastructure, rampant viral illness, incessant displacement, and entrapment. These unequivocally increase suicide risk and are linked to living under genocidal conditions—particularly entrapment.

At the end of the excerpt, I proclaim that, “Our universal language is prayer.” This is a passive wish. I know that suicide and genocide are connected, and that prayer is my most accessible form of action. Overall, my feelings of collective grief are amplified by the anticipatory grief of not knowing the fidelity with which the ceasefire plan is being implemented. I also express ambiguous loss over this same issue with the ceasefire but also not knowing where my Gazan colleagues are, and whether they are alive or murdered. Over so many years of activism against apartheid states, there used to be a disenfranchised grief I felt from the public being unaware of the Palestinian strife. Now, through this account, my grief is partially recognized, but this is not enough. For many Palestinians, grieving is ongoing, disenfranchised, ambiguous, anticipatory, and collective.

### 6.3. On Genocide and Suicide

Suicide must be also recognized as a direct and indirect product of structural colonial violence, i.e., apartheid, as a form of ongoing genocide. In the occupied Palestinian territories, and in the Gaza strip, such genocidal apartheid takes form as a linguicide of Arabic speakers (Zwisler 2021). Genocide tactics have targeted the decline of Arabic-speaking populations of all religious backgrounds for multiple decades. According to the Chair of the Palestinian Ministry of Mental Health, Dr. Samah Jabr, Gazans—who are majority Arabic speakers—face chronic traumatic stress disorder, not post-traumatic stress disorder, or PTSD (McKernan 2024). This type of stress results from ongoing violence exposure, and correspondingly increases suffering and hopelessness (Polanco-Roman and Miranda 2013; Taylor et al. 2011), which are two of the most pervasive risk factors for suicide across populations. Further, of the 2 million Gazans, more than half are under the age of 20, and are currently facing multiple adverse childhood experiences (Alakhras 2024), or ACEs, which are known risk factors of suicide; data collection to prove this is wanting (Jones et al. 2020). As a population, Gazans face military law, systematic starvation, lack of access to clean water, lack of health infrastructure, rampant viral illness, incessant displacement, and entrapment. These are unequivocally suicide risk factors and are linked to living under genocidal conditions—particularly entrapment (Taylor et al. 2011).

At its core, the very definition of suicide is violence that is self-targeted. Therefore, I believe it is imperative for researchers to understand the interlinked nature of genocide, linguicide, and suicide as violence syndemics. Previous researchers utilized ethnographic data to demonstrate evidence for similar theoretical perspectives on syndemics (Singer 2000).

## 7. Discussion

In this autoethnography, my grief accounts serve a greater purpose than to analyze my lived experience; in disseminating this work, it is a direct form of health communication relevant to the community. Through my storytelling and analysis, I elaborated on my lived experience with supporting citations from research literature on the concepts of disenfranchised grief, ambiguous loss, anticipatory grief, secondary loss, and collective grief. By doing so, I aimed to convey that grief is not static but rather an iterative process that involves ongoing construction of meaning. My personal narratives invite an understanding of grief that is culturally specific to people who may identify as Muslim, biracial, non-binary, having bipolar, and being bilingual. Offering analysis to a creative writing account of my lived experiences allowed me to illuminate aspects of grief that may otherwise be missed in dominant social contexts. Adding a positionality statement to this autoethnography serves the purpose of exploring reflexivity in a more meaningful way for the purpose of conducting suicide research in majority-world communities like Muslims.

Furthermore, it is well known by federal funding agencies that insider researchers can improve the inclusion of study participants, strengthen methodology, and ultimately enhance the rigor and impact of studies. Researchers with embodied lived experience may further deepen intervention effectiveness as storytellers or influencers in community settings where fostering trust is paramount to investigating suicide. The National Institute of Mental Health (NIMH) promotes transformative research and “encourages broad, interdisciplinary thinking in the development of scientific initiatives and programs and presses for theoretical leaps in science” (NIMH Director’s Innovation Speaker Series: Transformative Research Requires Insider Researchers n.d.). Moreover, participating as a service recipient with lived experience may deepen scholars’ intervention effectiveness as storytellers or influencers in community settings where fostering trust is paramount to addressing suicide.

### 7.1. Rigor of Methodology

This autoethnography embodies the six elements of rigor—time, place, social circumstances, intimacy, language, and consensus—as described by Ngunjiri et al. (2010), to ensure a rich, contextualized, and trustworthy narrative (Time is described through my detailed reflection on certain key life stages, particularly my experiences before, during, and after my suicide attempt, with attention to the evolving sociopolitical climate in the U.S. surrounding Muslims post-9/11. Place is made explicit through my geographic location in turtle island, or the United States, while also acknowledging the symbolic space of being a racialized and religious minority navigating institutions like healthcare, education, and law enforcement. The social circumstances surrounding my narrative—such as systemic Islamophobia and postcolonial trauma—are interwoven to reflect the intersectional and multidimensional context of my lived experience. Intimacy is demonstrated in my emotional vulnerability and previously private aspects of my life, inviting readers into my spectrum of affective states. I draw attention to my knowledge of the Arabic language, intentionally blending scholarly discourse with cultural expressions and everyday speech. Lastly, consensus is reflected in the resonance between my story and broader findings in Muslim mental health literature, as well as through feedback from peers and partnering colleagues who share similar social identities and have affirmed the authenticity and relevance of my narrative in both our professional and personal conversations. Together, these six elements anchor my autoethnography in methodological rigor.

### 7.2. Future Directions

Collaborative autoethnography is a newer method developed over the past decade (Lassiter 2005). In a collaborative autoethnography, a group of individuals work on developing a manuscript in a team-based dynamic. The purpose of collaborating is to enhance the ethics and reach of the messages being communicated. This brings to understanding some greater social phenomena, i.e., suicidality, that occur in cultural environments, i.e., the U.S. Muslim community. Community Advisory Board (CAB) members who serve as collaborators are ideal for collaborative autoethnographies, not only for being insiders but also for developing trust in the overall research process. By combining the CAB and participant voices, in the process and analysis of data collection, individual-level narratives may be validated from a group-level perspective. Ultimately, this cross-validation enhances the overall generalizability of the data in the autoethnography.

Conducting any autoethnography—individual or collaborative—necessitates inquiry into positionality, ethics, and bias (Darwin Holmes 2020). These are interlinked and must be evaluated in the context of doing this work in a collaborative manner with community partners from CBOs. In other words, when developing manuscripts, the author will engage in questions regarding the following: Who is the audience? What is the objective for telling a particular story? How is the narrative composed to reflect self-awareness of biases in conducting suicide research from a lived experience standpoint? A central concern in each of these questions is how power and positionality is considered between the researcher, CBO partners, and the greater community at large. Narrative methodology approaches to suicide research have been used in the past and recently to uncover topics like shame and stigma (Sather 2024).

These delve into deeper constructions of what is defined as “ethical,” both in theory and in practice. Considering that the author is a self-identified survivor, it is necessary to establish a code of conduct for interacting with communities on the matter of suicide prevention research. Strategic group decision-making around what should and should not be included in manuscripts, and the level of detail they should go into, are important factors for dissemination in the greater community. Additionally, the CBO leaders may have ideas on how to promote the framing of these messages such that the lay community audience may be receptive to them. For example, CBOs can offer suggestions on how to address and elicit help-seeking behaviors in the context of such barriers to mental healthcare. CBO partners may consider developing data sovereignty agreements in their memoranda of understanding to reflect the dignity of decedents and Muslims afflicted by suicidal thoughts and behaviors. This is what makes a collaborative autoethnography essential, as it may pave the way for future transformative research that counteracts Orientalist narratives. This includes challenging the trope of *jihad* as an act of suicide for terrorism and inviting critical perspectives of ‘revolutionary’ versus ‘reactive’ suicide as posited by Huey Newton, co-founder of the Black Panther Party (Newton 1973). Furthermore, the dynamic of embodying lived experience while being an insider participant–researcher demands an ethical framework that challenges existing notions of objectivity and power dynamics in human subjects’ research, though this is beyond the scope of this manuscript and should be investigated through collaborative approaches with community leaders. Some potential topics to explore include the role of reciprocity, the value of vulnerability, ways to engage in co-production and co-dissemination of data, and overall decolonial approaches (Lenette et al. 2019). It is also important to note that positivist logic should be challenged during these research endeavors, including rigid notions of identity as fixed instead of fluid, and this implies a fluidity dynamic of insider status, too. The rich autoethnographic detail I offer here, however, is a limited “insider status” in that being an insider is achieved better through active dialogue with others in the community. It should be understood that there are multiple realities and lived experiences that are only understood through social situatedness, where opportunities to reveal further complexities and consciousness of experience emerge. Therefore, developing collaborative approaches, such as collaborative ethnographies, is one way to offer meaningful future directions that deepen this singular account.

Additionally, there may be textual analysis such as those of the *hadith*, or sayings, of Prophet Muhammad—peace be upon him. For example, the *hadith* at the beginning of this manuscript may be interpreted through a qualitative analysis. In stating the context of calamity befalling a person, the Prophet insinuates an intimacy with moments of helplessness or hopelessness. However, the believing Muslim is not one to fall into a state of despair, as this would undermine Merciful God. Instead, the saying goes that wishing for life “if life is better for me” and, in contrast, wishing for death “if death is better for me” suggests a passive suicidal death wish. Yet, as a believer who submits to the will of Allah—a *Muslim* (translated as “one who submits”)—always recognizes that if they are alive, it is only so because that is how Allah has their best interests in mind. A Muslim may, thus, enter a suicidal crisis knowing that even the Prophet experienced a year of sadness and grief in his life, yet despair was not the state of mind, heart, or soul. Instead, placing faith that what happens in the worldly life will be accounted for in the hereafter gives the Muslim a wider outlook on justice, recognizing that it is beyond the scope of this material world. In holding belief in a just afterlife, the believer submits and defers to The Almighty, and recognizes that worldly strife is temporary, just as is the nature of suicidality. In the future, therefore, scholarly research from Islamic sciences such as *Hadith*, and Quranic text, must be cited as indigenous spiritual sources of knowledge beyond those cited by the traditional academic study of suicide.

## 8. Conclusions

It is well-established that one of the best predictors of suicide is a prior history of attempts (Bostwick et al. 2016); thus, researchers with lived experience of suicidality may offer insights into gaps in current research approaches as well as unique insights into social dynamics. This autoethnography of grief concepts is a narrative methodology that promotes relatability and trust that can help inform strategies for conducting suicide research through participatory approaches in community settings. The autoethnography of myself as an investigator with suicidal lived experience from a majority-world community is an original contribution to research. Furthermore, expanding this methodology to a collaborative ethnography will be a more innovative and deeper way to be reflexive, purposeful, and meaningful in conducting suicide research through community-led participatory approaches.

“By having no illusions about the system, and by being ready to die, we begin to live.”

—Huey P. Newton

## Data Availability

The original contributions presented in this study are included in the article. Further inquiries can be directed to the author.

## Footnotes

1 “Spoons” is an informal term referencing the popular cultural concept of ‘spoon theory.’ It is a term commonly used by people with chronic illnesses or disabilities. The term refers to a daily quota of 10 spoons worth of energy, to be strategically divvied up by priority in terms of survivalism.
